# MCC950, a specific small molecule inhibitor of NLRP3 inflammasome attenuates colonic inflammation in spontaneous colitis mice

**DOI:** 10.1038/s41598-018-26775-w

**Published:** 2018-06-05

**Authors:** Agampodi Promoda Perera, Ruchira Fernando, Tanvi Shinde, Rohit Gundamaraju, Benjamin Southam, Sukhwinder Singh Sohal, Avril A. B. Robertson, Kate Schroder, Dale Kunde, Rajaraman Eri

**Affiliations:** 1 School of Health Sciences, University of Tasmania, Launceston, TAS Australia; 2 Department of Pathology, Launceston General Hospital, Launceston, TAS Australia; 3 Institute for Molecular Bioscience, University of Queensland, St Lucia, QLD Australia

## Abstract

MCC950 a potent, highly specific small molecule inhibitor of canonical and noncanonical activation of NLRP3 inflammasome has been evaluated in a multitude of NLRP3 driven inflammatory diseases. However, the effect of MCC950 on colonic inflammation has not yet been reported. In the present study we investigated the effect of MCC950 in a spontaneous chronic colitis mouse model Winnie, which mimics human ulcerative colitis. Oral administration of 40 mg/kg MCC950 commencing at Winnie week seven for three weeks significantly improved body weight gain, colon length, colon weight to body weight ratio, disease activity index and histopathological scores. MCC950 significantly suppressed release of proinflammatory cytokines IL-1β, IL-18, IL1-α, IFNγ, TNF-α, IL6, IL17, chemokine MIP1a and Nitric Oxide in colonic explants. Moreover, MCC950 resulted in a significant decrease of IL-1β release and activation of caspase-1 in colonic explants and macrophage cells isolated from Winnie. Complete inhibition with MCC950 in Winnie colonic explants shows, for the first time, the contribution of inflammatory effects resulting exclusively from canonical and noncanonical NLRP3 inflammasome activation in colitis. Taken together, our results illustrate the efficacy of MCC950 in the treatment of murine ulcerative colitis and provides avenue for a potential novel therapeutic agent for human inflammatory bowel diseases.

## Introduction

Inflammatory bowel disease (IBD) is a group of intestinal disorders characterised by inflammation of the gastrointestinal tract^1^. The two major types of IBD are Crohn’s disease and ulcerative colitis. Crohn’s disease causes inflammation in all parts of the intestinal tissue along the length of gastrointestinal tract while ulcerative colitis is restricted to the mucosa of the colon and rectum. Both diseases are characterised by a series of relapses and remissions and also increases the risk of colon cancer^2^. The aetiology and pathogenesis of IBD is still unclear. Emerging evidence support the hypothesis that the dynamic key players are dysbiosis in enteric microbiota, a dysfunctional epithelial barrier, and defective innate immunity^3^.

The innate immune response to cell stress or infection depends on receptors such as Toll-like receptors (TLRs) and Nod-like receptors (NLRs)^4,5^. In particular NLRP3 is one of the best characterized and is associated with inflammatory diseases^6,7^. The NLRP3 inflammasome is a cytoplasmic multimolecular platform composed of NLRP3 protein bound to an adaptor protein, apoptosis-associated speck-like protein containing a CARD (ASC) and procaspase-1. Activation of the inflammasome leads to proteolytic activation of caspase-1 triggering cleavage and subsequent secretion of proinflammatory cytokines IL-1β and IL-18^8^. Kayagaki *et al*.^9^ described a novel non-canonical pathway resulting in NLRP3 inflammasome activation. This pathway is via caspase-11, which is widely expressed in both hematopoietic and non-hematopoietic cells, including macrophages and epithelial cells. Caspase-11 is activated by cytosolic gram-negative bacteria leading to pyroptosis and IL-1α and HMGB1 release, and NLRP3 inflammasome assembly and maturation of IL-1β and IL-18^9^.

IL-1β cytokine levels are significantly altered in patients suffering from either acute or chronic gastrointestinal inflammation and have been additionally implicated in tumour angiogenesis, progression, and metastasis^10,11^. Many clinical studies show evidence of increased IL-1β secretion from colonic tissues and macrophages of IBD patients, correlating to the severity of disease^12–14^. Preclinical studies imply that IL-18 contributes the pathogenesis of colitis^15,16^. Moreover, IL-18 neutralization^17,18^ or pralnacasan inhibition of Caspase-1^19,20^ effectively reduced severity in murine colitis. These clinical findings suggest IL-1β and IL-18 play an important role in the pathogenesis of IBD.

A study by Bauer *et al*.^21^ found that Nlrp3-deficient mice were significantly protected from colitis in DSS-induced colitis mouse model^21^ suggesting that the blockade of NLRP3 inflammasome may serve as a potential target for the development of novel therapeutics for patients with colitis. However, current pharmacological modulators of NLRP3 inflammasome tested in experimental colitis are not specific to NLRP3 inflammasome and do not inactivate both canonical and noncanonical pathways^22^.

MCC950 is a potent highly specific small molecule inhibitor of both canonical and noncanonical activation of NLRP3 inflammasome. *In vivo*, MCC950 reduced IL-1β production and attenuated the severity of experimental autoimmune encephalomyelitis, an animal model of multiple sclerosis which is known to be aggravated by the NLRP3 inflammasome^23^. Inhibition of NLRP3 by MCC950 effectively rescued neonatal lethality in a mouse model of cryopyrin-associated periodic syndrome, a genetic disease caused by activating mutation in NLRP3. In agreement with cell profiling, MCC950 was not effective against an NLRP1 mutant highlighting the compounds specificity *in vivo*. The study provided a detailed pharmacokinetic profile of MCC950 but the mechanism of action was elusive; MCC950 did not affect K^+^ efflux, Ca^2+^ flux, NLRP3-NLRP3 or NLRP3-ASC interactions^23^. Further work by Primiano *et al*.^24^, dismissed other likely targets of MCC950 such as GST Omega 1-1^25^, SUR1, SUR2a and SUR2b. Moreover MCC950 did not target cellular proteins involved in the activation of NLRP3 inflammasome such as caspase-1, SYK, JNK, GPR40, and GPR120^24^. Only very weak off-target activity was identified through multiple commercially available screening panels (Eurofins Cerep, DiscoverX, Reaction Biology, Carna Biosciences, WuXi AppTec)^24^. In 2016 two independent studies discovered NEK7, a serine-threonine kinase, as an upstream regulator of NLRP3 inflammasome activation^26,27^. This major discovery of a new inflammasome component, revealed a potential therapeutic target for the inhibitory mechanism of sulfonylurea molecules such as MCC950 and glyburide. We and others hypothesise that MCC950 and glyburides target of inhibition could be the NEK7-NLRP3 interaction^28^.

MCC950 is the most specific and well characterised NLRP3 inhibitor known to date and has been tested in a diverse array of NLRP3 engaged inflammatory diseases. MCC950 shows promising therapeutic potential for reducing crystal-induced kidney fibrosis in mice^29^, reversing inflammation and blood pressure in a hypertension mouse model^30^, in valosin-containing protein associated disease^31^ and decreasing inflammation associated with pathogenic Influenza A Virus^32^. Temporal administration of MCC950 was able to reduce lung inflammation and cellular influx^33^. However, MCC950 was not effective in reducing angiotensin II induced hypertension^34^ and in the treatment of acute procedural inflammation in burn-injured mice^35^. This was however due to the limited role of NLRP3 inflammasome in these disease models.

Recently MCC950 was recommended as an ideal therapeutic candidate for the selective inhibition of NLRP3 in colitis^22^. Pellegrini *et al*.^36^ suggested MCC950 treatment will define anti-inflammatory effects resulting exclusively from inhibition of canonical and noncanonical NLRP3 inflammasome activation in colitis^36^. At present, the majority of available studies on the efficacy of NLRP3 inhibitors have used dextran sulfate sodium (DSS) induced acute colitis as the experimental model of ulcerative colitis^22^. The DSS colitis model is very established due to its rapidity, reproducibility and controllability. The DSS chemical exerts a toxic effect on colonic epithelium leading to a leaky tight junction and bacterial translocation^37^. Therefore it is reflective more of an acute injury than an inflammatory disease^38^. In addition DSS induced colitis development does not involve the T and B cell immunity which is unlike human ulcerative colitis^39^. Due to these limitation in DSS induced colitis there is a great need for clinically relevant spontaneous colitis murine models which resembles human disease for understanding the inflammatory immune process of ulcerative colitis.

In this study we have used the spontaneous chronic colitis mouse model Winnie which develops spontaneous distal intestinal inflammation as early as 6 weeks of age and progresses over time to severe colitis by 16 weeks^40,41^. Chronic colitis in Winnie is caused by a primary epithelial cell defect due to a point mutation in the *Muc2* gene resulting in aberrant mucin-2 biosynthesis leading to endoplasmic reticulum stress in intestinal goblet cells and reduced secretion of mucus which is very similar to active ulcerative colitis in humans^42,43^. Winnie mice display symptoms of diarrhoea, ulcerations, rectal bleeding and pain at different stages of colitis similar to human disease. Extensive studies done in Winnie has proven it to be the best available murine model to study human chronic colitis and its pathogenesis^44–46^. The aim of this study was to investigate the therapeutic effect of MCC950 on Winnie and our results show a significant reduction of colitis.

## Results

### MCC950 inhibits the activation of NLRP3 inflammasome in mouse macrophages

IL-1β is processed from the inactive cytoplasmic precursor pro-IL-1β which has to be cleaved by caspase-1 to produce the mature active form. We examined the ability of MCC950 to inhibit the activation of pro-IL-1β by inhibiting the activation of NLRP3 inflammasome. For our initial experiment we used a concentration of 0.01 µM of MCC950 which is close to 0.0075 µM, the half-maximal inhibitory concentration (IC_50_) of MCC950 in bone marrow derived macrophages (BMDM) of C57BL/6 mice. We isolated BMDM, Intraperitoneal (IP),mesenteric lymph node (MLN) and lamina propria mononuclear cell (LPMC) murine macrophages from Winnie and C57BL/6 mice. We could not isolate enough MLN and LPMC from C57BL/6 for inflammasome activation experiments.

**Figure 1:**
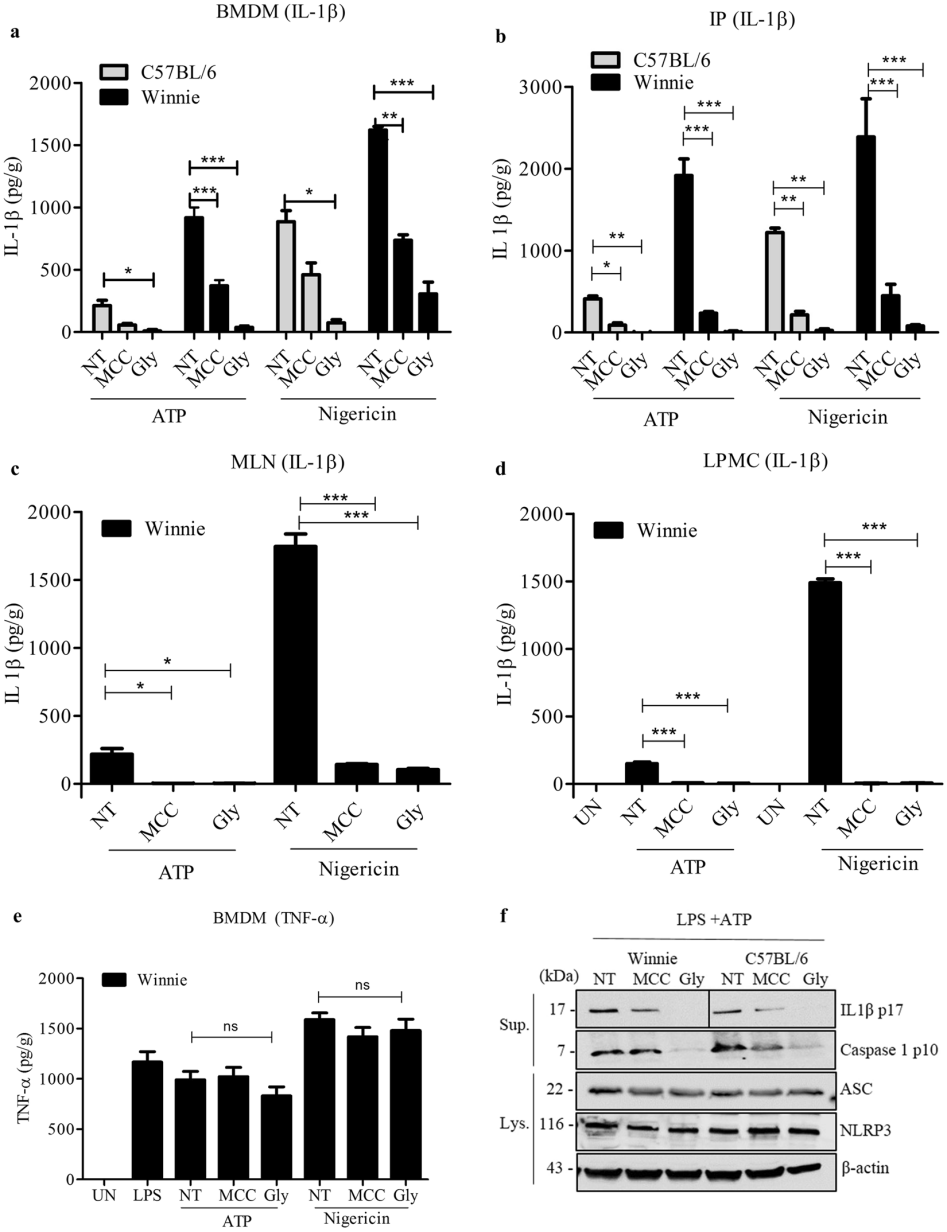
The effect of MCC950 on NLRP3 inflammasome activation in murine macrophages. Production of IL-1β (**a**) C57BL/6 and Winnie BMDMs (**b**) C57BL/6 and Winnie IP Macrophages (**c**) Winnie MLNs (**d**) Winnie LPMCs. Unprimed (UN), primed with 10 ng/ml LPS and treated with MCC950 (MCC) (**a**–**c**) 0.01 µM, (**d**) 1 µM) and Glyburide (Gly) (200 µM) and stimulated with ATP and Nigericin as measured by ELISA. (**e**) Production of TNF-α in Winnie BMDM supernatants treated with MCC950 0.01 µM and glyburide 200 µM and stimulated with ATP and Nigericin as measured by ELISA. Data are expressed as the mean ± sem of three independent experiments carried out in duplicates. *P < 0.05, **P < 0.01, ***P < 0.001 (one-way ANOVA with Tukey’s post-hoc test). (**f**) Western blots of cell lysates and supernatants from C57BL/6 and Winnie BMDMs primed with 10 ng/ml LPS and treated with MCC950 (0.01 µM) or glyburide (200 µM) and stimulated with ATP. These results are representative of three independent experiments.

Our results showed IL1-β release was markedly increased in the macrophages of Winnie mice compared with C57BL/6 upon LPS treatment. Cells were then pre-treated with MCC950 or glyburide and then stimulated with the NLRP3 agonists ATP or the ionophore nigericin. Treating cells with 0.01 µM of MCC950 and 200 µM glyburide significantly inhibited the release of IL-1β in BMDMs (Fig. 1a), IPs (Fig. 1b) and MLNs (Fig. 1c). Complete inhibition of IL-1β was observed in LPMCs treated with MCC950 at 1 µM and stimulated with specific NLRP3 stimulants ATP and Nigericin (Fig. 1d). LPS-dependent TNF-α secretion was not impaired by MCC950 in BMDMs (Fig. 1e), which demonstrates that the inhibition of IL-1β secretion was specific. To investigate the potential cytotoxicity effect of MCC950, we performed the alamarBlue® cell viability assay. The results show that the there is no cytotoxic effects on Winnie BMDM cells against MCC950 at 0.001 µM–1 µM (Fig. S1).

The amount of active caspase-1 p10 was reduced in supernatants from MCC950 treated Winnie and Wild type BMDMs (Fig. 1f), suggesting that MCC950 inhibits the activation of caspase-1 by NLRP3. Correspondingly, the processing of IL-1β was inhibited by MCC950. Similarly, treatment with glyburide inhibited caspase-1 activation and IL-1β processing. Western blot analysis revealed that expression of the inflammasome complex proteins such as NLRP3 and ASC were not changed during treatment with MCC950 (Fig. 1f).

### MCC950 inhibits the activation of NLRP3 inflammasome in colonic explants

**Figure 2:**
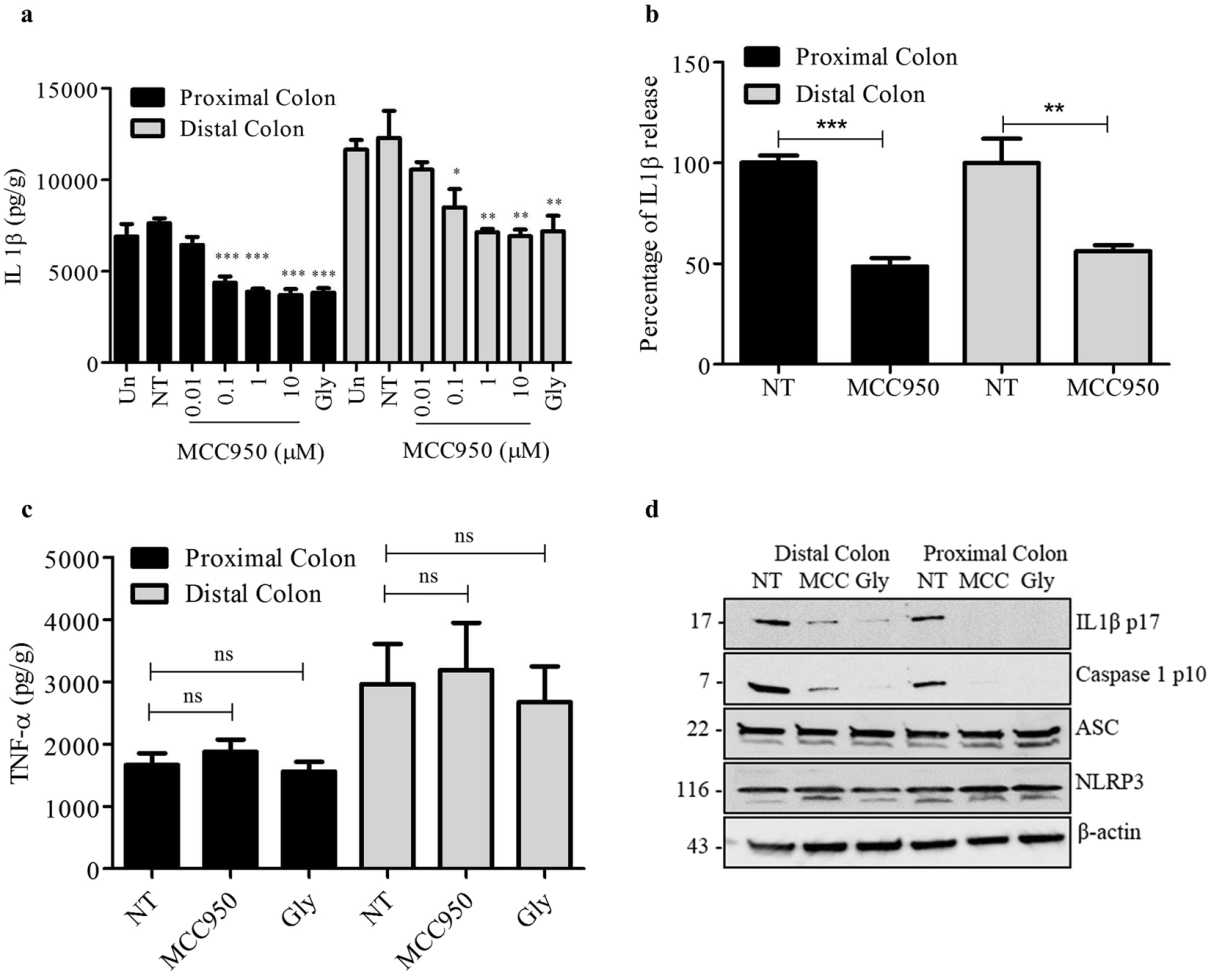
MCC950 inhibits NLRP3 inflammasome activation in colonic explants. (**a**) Production of IL-1β from Winnie proximal and distal colons stimulated with 10 ng/ml LPS and treated with MCC950 (MCC) (0.001–10 µM) and Glyburide (Gly) 200 µM as measured by ELISA. (**b**)Percentage of IL-1β release of Winnie proximal and distal colons stimulated with LPS no treatment compared to treated with MCC950 (10 µM) as measured by ELISA. (**c**) Production of TNF-α for proximal and distal colonic explant supernatants treated with MCC950 (1 µM) and glyburide 200 µM as measured by ELISA. Data are expressed as the mean ± SEM of five independent experiments carried out in duplicates. *P < 0.05, **P < 0.01, ***P < 0.001 (one-way ANOVA with Tukey’s post-hoc test). (**d**) Western blots of tissue lysates and supernatants from proximal and distal colons stimulated with 10 ng/ml LPS and treated with MCC950 (1 µM) or glyburide (200 µM). These results are representative of three independent experiments.

To further explore the effect of MCC950 on NLRP3 inflammasome activation in colitis, we investigated the release of IL-1β in treated Winnie colonic tissue explants by ELISA. Non treated Winnie distal colon produces 12, 307 pg/g which is comparatively higher than the 7623.5 pg/g released by proximal colon tissue. MCC950 exhibited a concentration-dependent inhibition of IL-1β secretion from LPS treated Winnie proximal and distal colon explant tissue (Fig. 2a). Treating colon explant with 10 µM concentration of MCC950 significantly reduced the release of IL-1β in proximal colon to 48.6% P < 0.001 and the distal colon to 56.2% P < 0.01 (Fig. 2b). LPS-dependent tumour necrosis factor-α (TNF-α) secretion was not impaired by 1 µM MCC950 and 200 µM glyburide (Fig. 2c) which demonstrates that the inhibition of IL-1β secretion was specific. The western blot analysis showed that 1 µM MCC950 and 200 µM glyburide significantly inhibited the activation of caspase-1 in to the cleaved form caspase-1 p10. Correspondingly, 1 µM MCC950 and 200 µM glyburide suppressed the processing of proIL-1β to mature IL-1β (Fig. 2d).

### Oral administration of MCC950 attenuates colonic inflammation in Winnie

**Figure 3:**
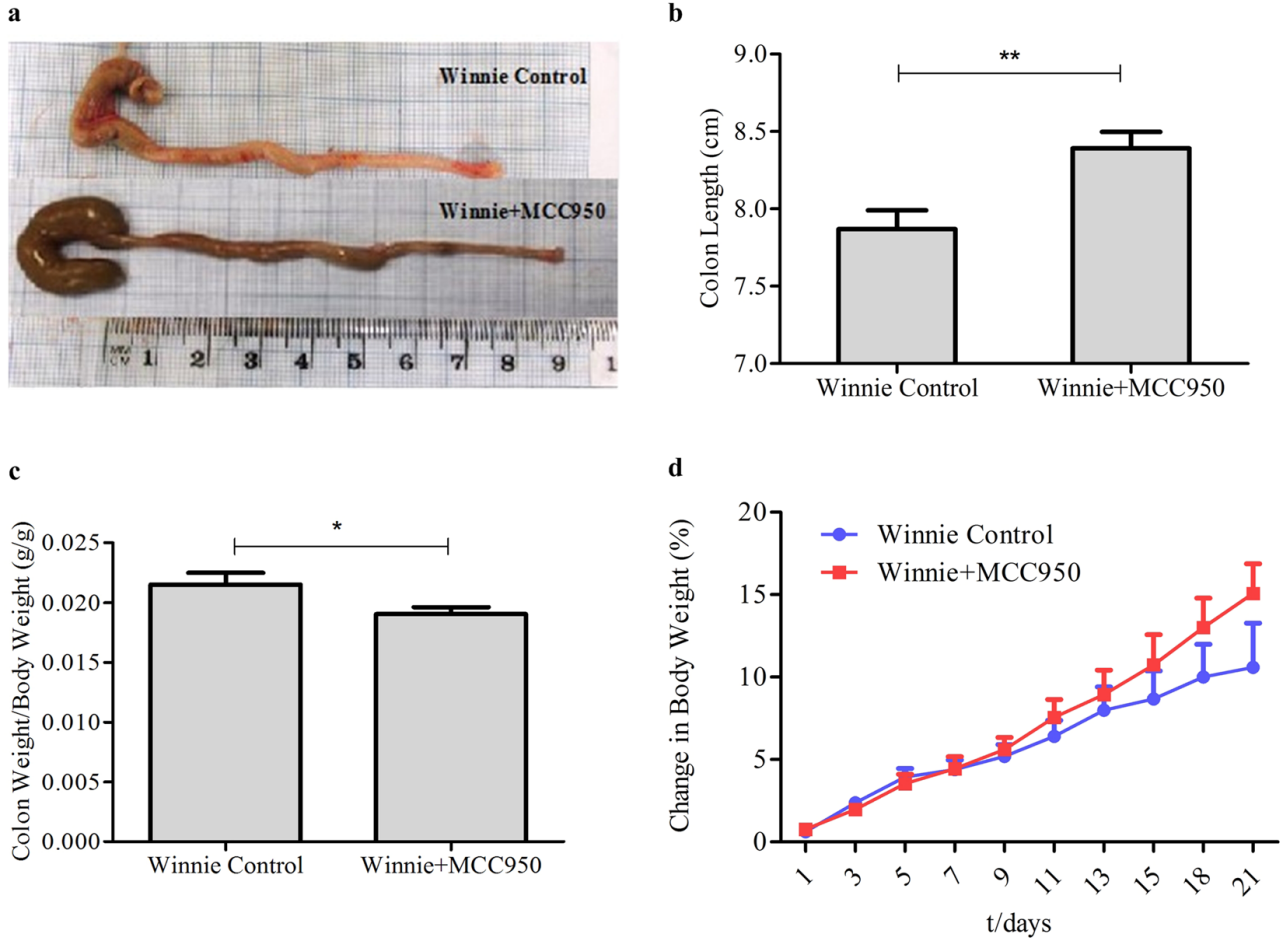
Effect of MCC950 on Winnie. Winnie at 10 week were weighed on the day of termination. Lengths of the freshly removed colons from each group were measured from ileocecal junction to rectum. The weight of the colons after removing luminal content was recorded. (**a**) Macroscopic appearances and (**b**) Colon Length for each group. (**c**) Ratio of colon weight over body weight. Data are expressed as the mean ± SEM (n = 10 per group) *P < 0.05, **P < 0.01 (two-tailed Student’s t test). (**d**) Body weight of mice was measured every 3 days and presented as a percentage of their initial weight. Data are represented as means ± SEM (n = 10 per group) repeated-measures analysis of variance (ANOVA).

The macrophage cell *in vitro* data and colon explant data suggested that MCC950 effectively inhibited NLRP3 inflammasome assembly and may be key to controlling colitis. To assess this, we examined the effect of MCC950 (40 mg/kg) in seven week old Winnie mice, administered orally for three weeks, in a spontaneous colitis mouse model Winnie. Throughout the experiment, mice were monitored for the clinical symptoms of colitis. Macroscopic observation of the 10 week control Winnie colon at experimental termination showed the colons to be visibly inflamed with shortening and thickening of colon wall with enlarged mesenteric lymph nodes when compared to MCC950 treated 10 week Winnie in (Fig. 3a). The mean colon length of the MCC950 treated group 8.390 ± 0.1080 was significantly (*P* < 0.01) longer than the mean colon length of the control group 7.870 ± 0.01212 (Fig. 3b).

MCC950 40 mg/kg prevented the shortening of the colon which is positively related to the severity of colitis. Wet colon weight, an indicator of intestinal oedema and inflammation, was presented as the ratio of colon weight over body weight (g/g). The untreated colitis group showed the highest relative weight 0.02152 ± 0.00096. MCC950 treatment significantly (*P* < 0.05) reduced the mean to 0.01902 ± 0.00060 (Fig. 3c).

**Figure 4:**
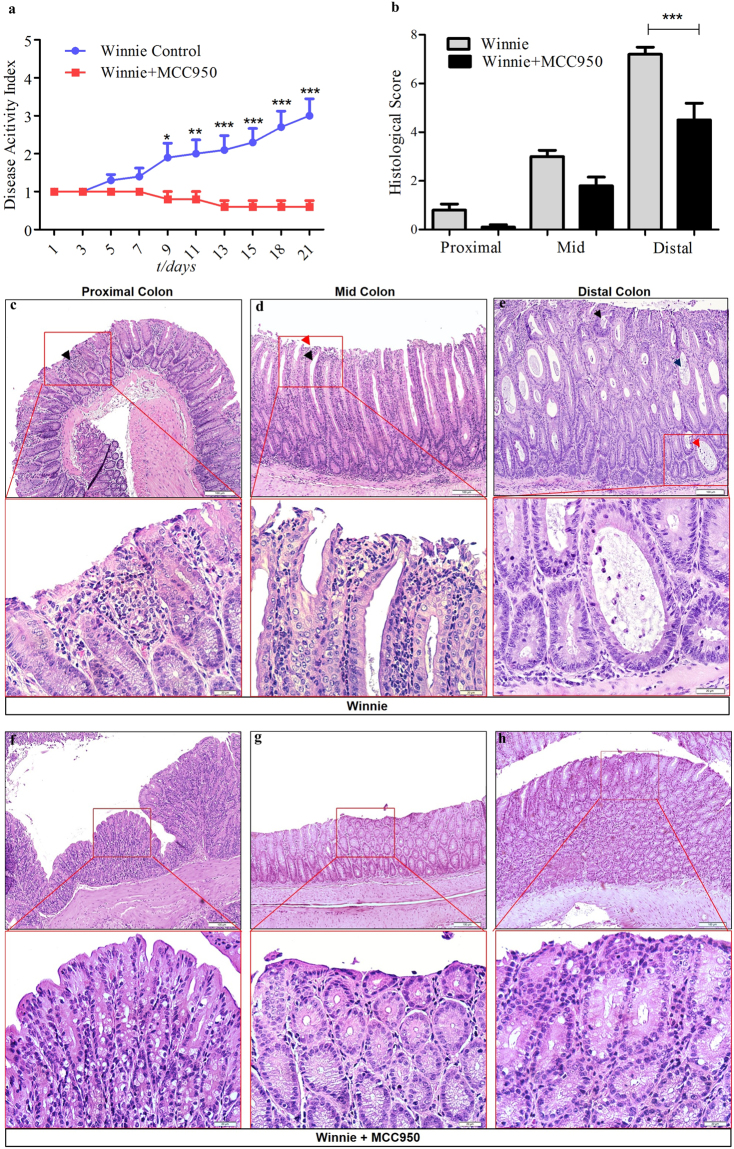
MCC950 treatment improves colitis in 10 week old Winnie. (**a**) Disease activity index. (**b**) Comparison of summed inflammation scores between control and treatment Winnie mice. PC, proximal colon, MC, middle colon, DC, Distal Colon. Data are represented as means ± SEM (n = 10 per group) *P < 0.05, **P < 0.01, ***P < 0.001 (one-way ANOVA with Tukey’s post-hoc test). (**c–e**) Representative Winnie control proximal, middle and distal colon sections stained with hematoxylin and eosin at 100x and 400x. (**c**) Lamina propria inflammatory cell infiltrates (black arrow). (**d**) Epithelial surface damage (red arrow), goblet cell loss (black arrow). (**e**) Crypt abscesses with neutrophils in the lumen and nearly intact epithelium (red arrow) or damaged epithelium and complete crypt loss (blue arrow) and crypt architectural distortion (black arrow). (**f**–**h**) Representative MCC950 treated Winnie proximal, middle and distal colon sections stained with hematoxylin and eosin at 100x and 400x.

To determine the therapeutic potential of MCC950 on colitis we characterised the control and treatment group by clinical parameters such as percentage of body weight gain and disease activity index (DAI) which is an average score of stool consistency, and blood in stool. As shown in (Fig. 3d) in contrast to the control mice which gained 10.59% body weight over 21 days, MCC950 treated mice showed an average of 15.07% body weight increase. However the body weight increase was not statistically significant between the control and treatment groups. MCC950 40 mg/kg significantly improved the DAI as early as the 9^th^ day of treatment showing the highest significance at day 21 at *P* < 0.001(Fig. 4a). The increase in body weight correlated with a significant decrease in DAI for the MCC950 treated group. The relationship between these two clinical parameters was significant at P < 0.0001 with a spearman’s correlation of r = 0.9342.

Histological analysis showed infiltration of neutrophils (Fig. 4c), severe surface epithelial damage (Fig. 4d), crypt abscesses (Fig. 4e), distortion of crypt architecture (Fig. 4e), and complete loss of cypts (Fig. 4e), particularly in the distal colon of colitis mice compared to MCC950 treated mice (Fig. 4f–h). The results of standard pathological examination of mouse colon reflected in a histological score showed much improvement in pathological changes in mice treated with 40 mg/kg of MCC950 which was statistically significant for distal colon at a *P* < 0.001. The comparative histological score of the mid colon was lower in the MCC950 group, however it was not statistically significant. These data reveal that MCC950 improves clinical and histological changes in the colon associated with spontaneous colitis.

### Oral administration of MCC950 suppresses colonic IL-1β and IL18 expression in Winnie

**Figure 5:**
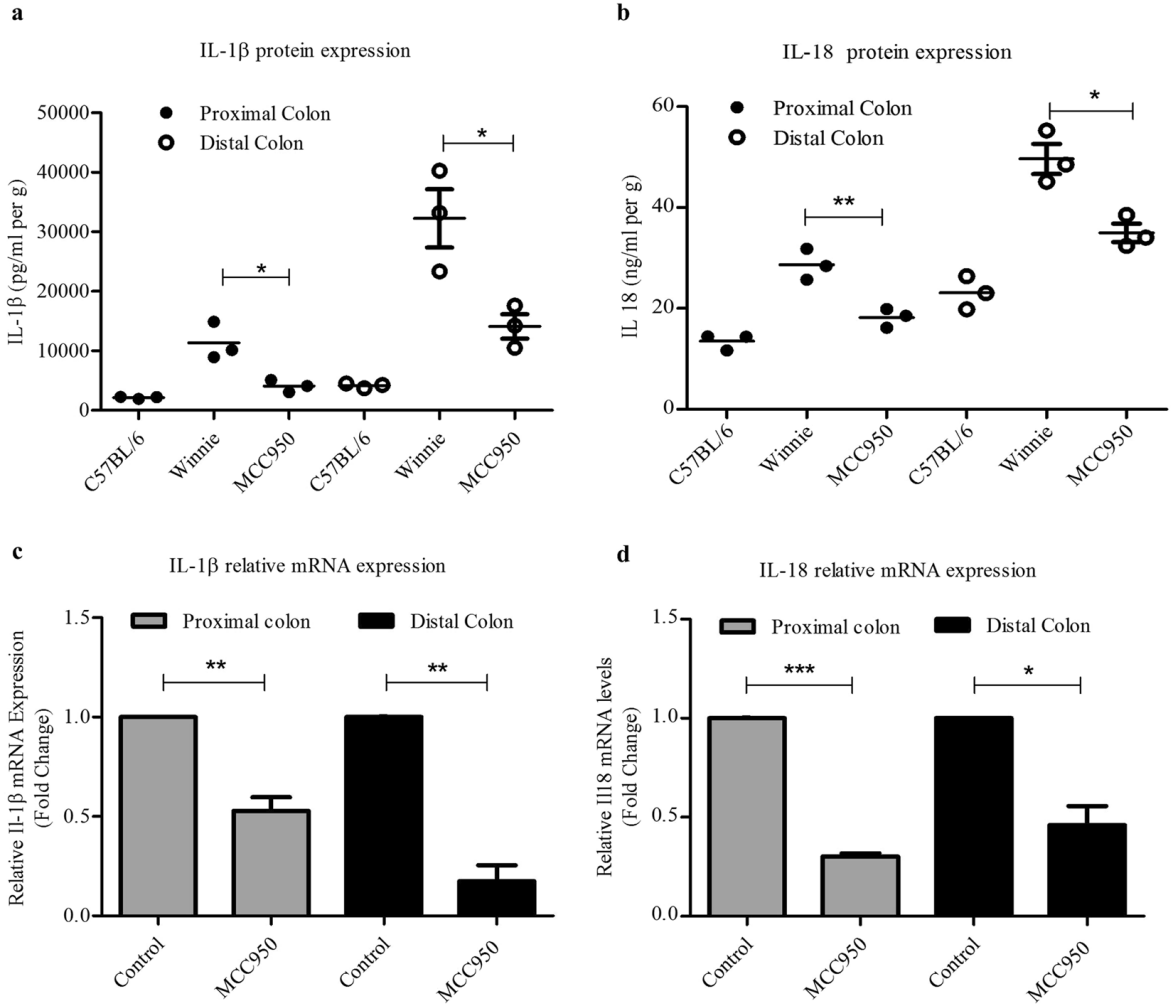
MCC950 suppressed NLRP3 activated proinflammatory cytokine levels in colon explant of Winnie mice. Protein levels of cytokines (**a**) IL-1β (**b**) IL18 in proximal and distal colon explant supernatants as determined by Bio-plex. Data presented as means ± SEM (n = 3 per group) *P < 0.05, **P < 0.01 (two-tailed Student’s t test). The mean values of fold change in mRNA expression levels for (**c**) *IL-1β* (**d**) *IL18* in MCC950 treated Winnie proximal and distal colon tissue are shown relative to the untreated Winnie proximal and distal control samples respectively. Both control and treated values were normalised to those of the internal control *Gapdh*, with treated values representing the fold change relative to that of controls, which was converted to 1. Data are expressed as the mean ± SEM (n = 4 per group) *P < 0.05, **P < 0.01 ***P < 0.001 (one sample t test).

To determine the effect of MCC950 on IL-1β and IL18 cytokine production in colitis mice, cytokine expression in colonic tissue at both mRNA and protein levels in both groups were measured. MCC950 treatment was able to significantly (*P* < 0.05) suppress IL-1β cytokine in proximal and distal colon compared to control group colons (Fig. 5a). The suppression of IL18 was at a significant level at proximal colon at *P* < 0.01 and distal colon at *P* < 0.05 (Fig. 5b). Total RNA of colons were extracted and analysed for cytokine mRNA expression using quantitative real time PCR method. MCC950 treatment was able to significantly suppress IL-1β mRNA relative expression in proximal colon to 0.5277 (*P* < 0.01) and distal colon to 0.1749 (*P* < 0.01) (Fig. 5c) and IL18 mRNA relative expression in proximal colon to 0.3016 (*P* < 0.001) and distal colon to 0.4606 (*P* < 0.01) (Fig. 5d).

### Oral administration of MCC950 reduces colonic proinflammatory cytokines

**Figure 6:**
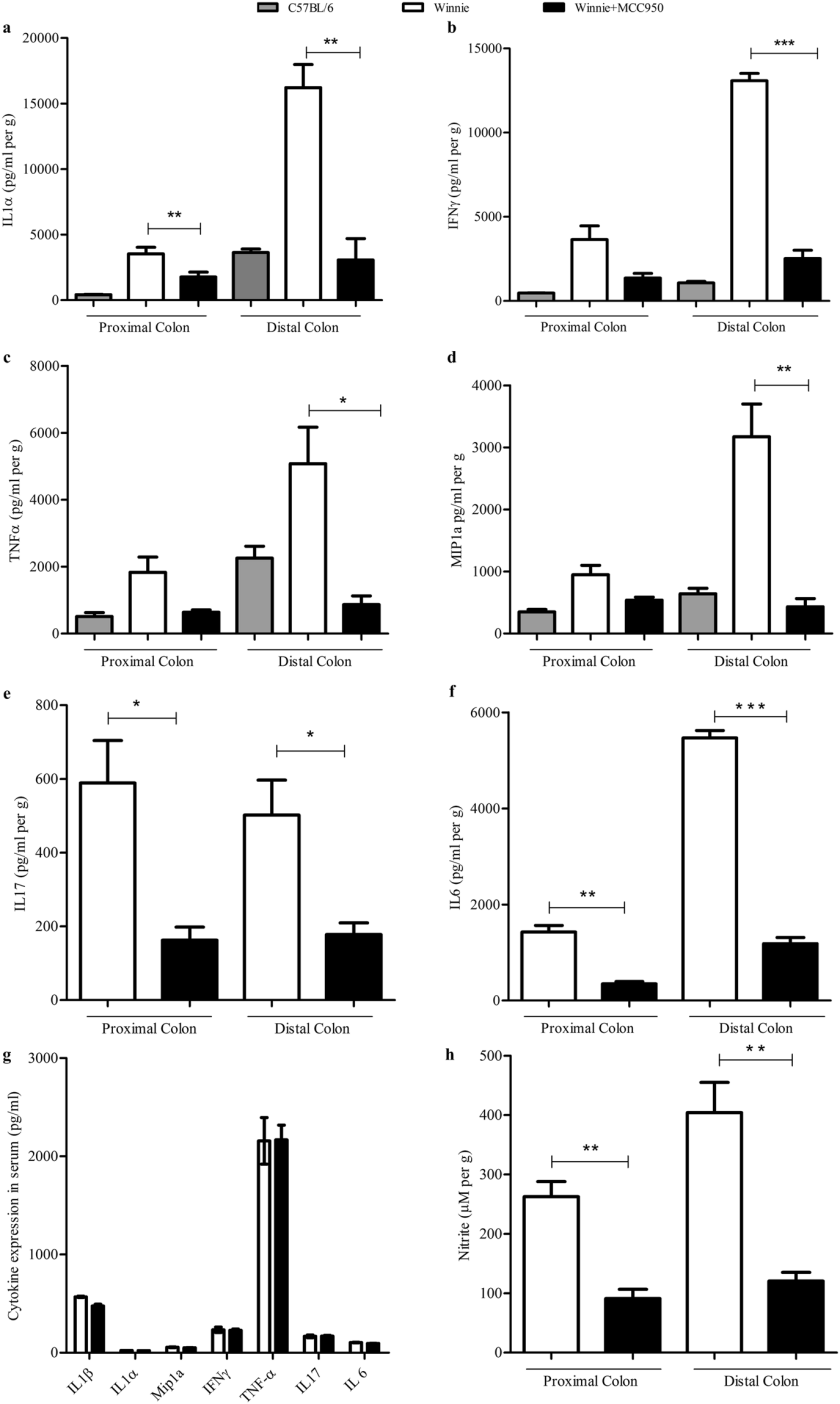
MCC950 suppressed proinflammatory cytokine and chemokine production in colon tissues but not in blood serum. Protein levels of cytokines including (**a**) IL1α (**b**) MIP1a (**c**) IL17 (**d**) IFNγ (**e**) TNF-α in explant supernatants (**f**) IL-1β, IL-1α, MIP1a, IL17, IFNγ, and TNF-α in blood serum were determined by Bio-plex. Data are presented as means ± SEM (n = 3) *P < 0.05, **P < 0.01, ***P < 0.001 (two-tailed Student’s t test).

While the mucosal explants isolated from Winnie colon control group actively secreted multiple proinflammatory cytokines (IL1-α, IFNγ, TNF-α, IL17 and IL6), chemokine (MIP1a) and Nitric Oxide during 24-hour culture, MCC950 treatment effectively suppressed their release (Fig. 6).

IL1-α was highly suppressed in MCC950 treated proximal and distal colons (*P* < 0.01) (Fig. 6a). IFNγ was suppressed in MCC950 treated proximal colon, however it was not statistically significant. IFNγ was strikingly suppressed in the distal colon (*P* < 0.001) (Fig. 6b). TNF-α was suppressed in MCC950 treated proximal colon however it was not statistically significant. In the distal colon TNF-α was significantly suppressed (*P* < 0.001) (Fig. 6c). MIP1a also known as the CCL3 chemokine was suppressed in MCC950 treated proximal colon however it was not statistically significant. In the distal colon MIP1a was effectively suppressed (*P* < 0.01) (Fig. 6d). IL17 was highly suppressed in MCC950 treated proximal and distal colons (*P* < 0.05) (Fig. 6e). IL6 was also highly suppressed in MCC950 treated proximal and distal colons (*P* < 0.01) (Fig. 6f). Interestingly MCC950 had no effect in blood plasma proinflammatory cytokines (IL1-β, IL1-α, TNF-α, IFNγ, IL17 and IL6) and chemokine (MIP1a) levels when compared to control group at the termination day at 24 hours after final treatment (Fig. 6g). Nitrite was measured as an index of Nitric Oxide generation and was highly suppressed in MCC950 treated proximal and distal colons (*P* < 0.01) (Fig. 6h).

### MCC950 and Glyburide do not target NEK7-NLRP3 interaction

**Figure 7:**
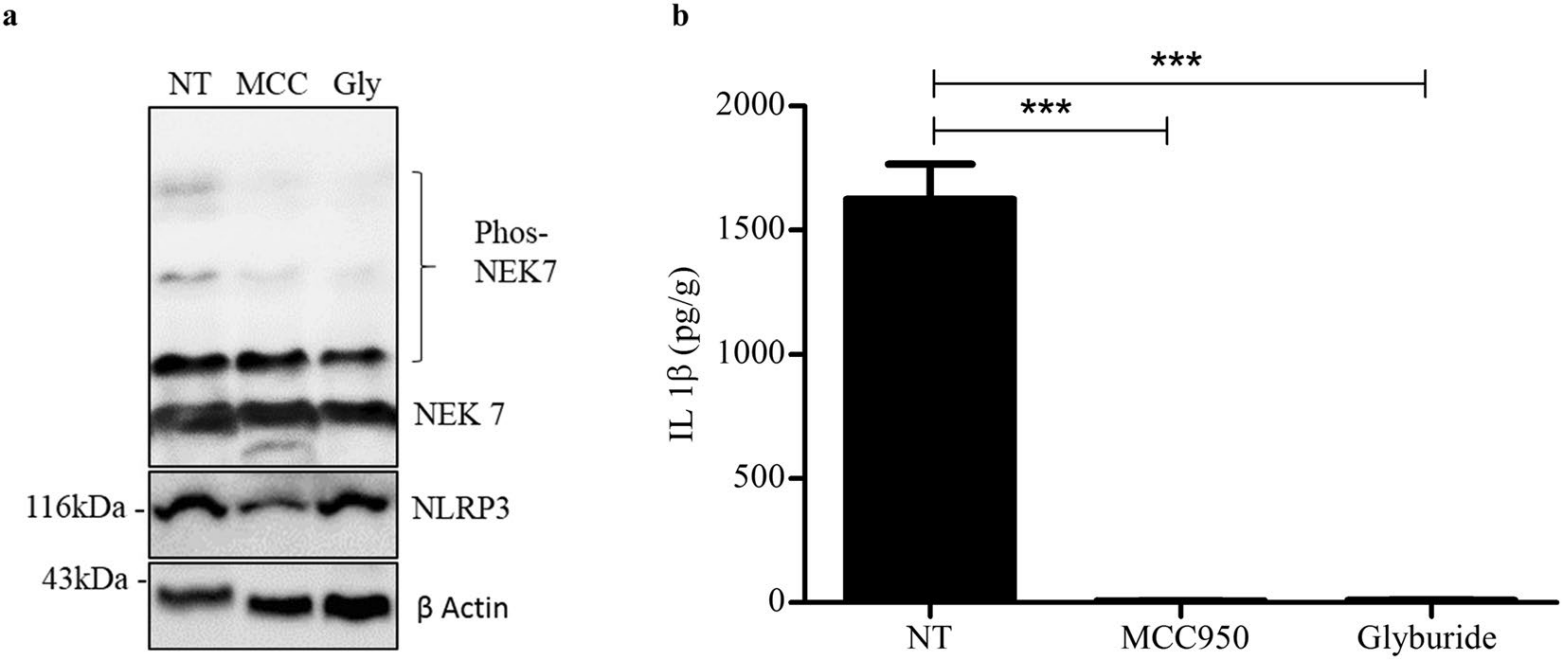
J774A.1 cells were primed with 100 ng/ml LPS and inhibited with 1 µM MCC950 or 200 µM Glyburide then stimulated with 5 mM ATP. (**a**) The phosphorylation state of NEK7 was analysed using Phos-tag SDS-PAGE. These results are representative of three independent experiments. (**b**) ELISA analysis of IL-1β in the culture supernatant of J774A.1 cells treated as in (**a**). Data are expressed as the mean ± sem of three independent experiments carried out in duplicates. ***P < 0.001 (one-way ANOVA with Tukey’s post-hoc test).

NEK7 undergoes phosphorylation during the interaction with NLRP3 component and the level of phosphorylation correlates to the activation of the inflammasome^27^. To examine the possibility of MCC950 and glyburide targeting the NEK7-NLRP3 interaction, we assessed the phosphorylation component of NEK7. We primed the murine macrophage cell line J744A.1 with LPS and applied the inhibitors and activated the NLRP3 inflammasome with ATP and analysed the level of NEK7 phosphorylation using Phos-tag SDS-PAGE. The Phos-tag SDS-PAGE blot analysis showed that the level of phosphorylated NEK7 band was the same size for MCC950 and glyburide and the untreated sample (Fig. 7a). This result concludes that both the inhibitors did not react by inhibiting the NEK7-NLRP3 interaction. The IL-1β levels were suppressed by the inhibitors, recognizing that the inhibitors were effective (Fig. 7b).

## Discussion

Our study describes for the first time the oral administration of MCC950 in the spontaneous colitis murine model Winnie. The most salient finding of the study is that MCC950 decreased the severity of chronic colitis in Winnie mice by specific inhibition of NLRP3 inflammasome activation. In addition MCC950 treatment significantly decreased the expression of proinflammatory cytokines IL-1β and IL-18 at mRNA and protein level and the associated release of proinflammatory cytokines and chemokine in Winnie colonic tissue. Moreover, MCC950 resulted in a significant decrease of IL-1β and active caspase-1 in Winnie explants and *in vitro* macrophage cells isolated from Winnie mice. Our data shows that the NLRP3 inflammasome plays a negative role in the chronic phase of ulcerative colitis in Winnie. Our results collectively suggest that MCC950 acts as an effective therapeutic compound for the treatment of murine ulcerative colitis.

Our results show that IL1β is released more in the Winnie distal colon when compared to the proximal colon. This explains why MCC950 is more effective in the distal colon and shows more improvement with MCC950. We further analyzed the Winnie explant IL-1β ELISA data treated at 10 µM, as this high dose will ensure complete efficacy in NLRP3 inflammsome inhibition even in the presence of explant serum. Our results show that the NLRP3 specific inhibitor MCC950 at 10 µM inhibited the release of IL-1β in proximal colon by 51.4% and the distal colon by 43.7%. This is an important finding as this indicates the percentage of canonical and non-canonical NLRP3 inflammasome contribution to the overall IL-1β in the spontaneous colitis colon of Winnie mice. High NLRP3 expression has been found in the ulcerated colonic tissue and in the colon of mice with acute and chronic colitis^47^. Accumulating evidence support a pro-inflammatory contribution of NLRP3 to colitis pathology^48,49^. It is reported that blockage of IL-1β^50^ or neutralization of IL-18^18,51^ reduces intestinal inflammation. These findings support the detrimental role of the NLRP3 inflammasome in the development of spontaneous colitis in Winnie which is supported by both DSS model^21^ and IL-10^−/−^ model of colitis^52^. The explant results show that selective inhibition of the NLRP3-inflammasome with MCC950 dose dependently reduces activated IL-1β secretion from colon tissue simulating a clinically feasible treatment regimen.

A study by Bauer *et al*.^21^ has proved that the mechanism of NLRP3/ASC/caspase-1-mediated activation of proinflammatory IL1-β and IL18 is essential for experimental colitis^21^. The western blot results of the *in vitro* experiments showed that treatment with MCC950 significantly reduced the active caspase-1 p10 and IL-1β in BMDMs and colon explants suggesting that MCC950 inhibits the activation of NLRP3 inflammasome in the colitis model. Similarly, treatment with glyburide inhibited caspase-1 activation and IL-1β processing as seen in protein quantification assays.

For our *in vitro* experiments we chose to use primary macrophages because NLRP3 and ASC complex is mainly expressed in these innate immune cells^53^ and they have a vigorous inflammasome activation and IL-1β production. The primary macrophage cells isolated from Winnie had a higher secretion of IL-1β in comparison to C57BL/6 indicating active colitis in Winnie. Similarly it is seen in murine DSS models^54^ and human colitis patients^14^ where active colitis and its severity has been correlated to high levels of IL-1β secreted by activated macrophages. Intestinal inflammation is associated with infiltration of macrophages into the colon lamina propria where a variety of inflammatory cytokines are produced in response to intestinal microbes. A very recent research in gut microbiota has shown that NLRP3 is absent in the epithelium but present in the deeper residing lamina propria monoculear cells^55^. Our *in vitro* results on LPMCs show complete inhibition of IL-1β by 1 µM of MCC950, which explains how MCC950 attenuated colonic inflammation in our *in vitro* spontaneous colitis model Winnie.

For *in vitro* experiments apart from MCC950 we have also used glyburide, another specific inhibitor of NLRP3 inflammasome^56^. A recent study investigated the effect of glyburide in IL10^−/−^ spontaneous Crohn’s disease mouse model^57^. Very similar to our results with MCC950 in Winnie mice, glyburide effectively suppressed NLRP3 inflammasome activation in IL-10^−/−^ mice, leading to attenuation and prevention of colitis. Moreover, glyburide also effectively inhibited the release of proinflammatory cytokines and chemokines in a similar manner to MCC950 effect in the Winnie model. Our *in vitro* results with glyburide shows that it is effective in inhibiting NLRP3 inflammasome in a spontaneous ulcerative colitis mouse model. Further *in vivo* studies should be conducted to evaluate the potency of glyburide in Winnie. However in comparison to glyburide, MCCC950 has superior pharmacological characteristics such as higher potency (7.5 nM), oral bioavailability (68%), temporal application and no known side effects^23^. Current experimental NLRP3 inhibitors of colitis are applied parenterally^58,59^, however in our study we have shown effective oral administration with MCC950 which is more clinically desirable than parenteral application.

In our study MCC950 treatment significantly improved clinical parameters of body weight gain, colon length, bloody stool and stool consistency. Similarly histopathological findings supported that MCC950 protected Winnie mice from surface epithelial erosion, inflammatory cell infiltration, loss of goblet cells and disruption of crypt architecture. These findings indicate oral administration of specific NLRP3 inhibitor MCC950 at 40 mg/kg decreased the severity of spontaneous chronic colitis in Winnie mice.

Ulcerative colitis is characterized by a disturbed balance between regulatory and effector cells which mainly implicates effector T cells (Th1 and Th2), regulatory T cells (Tregs) and Th17 cells^60^. The intestinal inflammation is characterized by a Th1 and Th17-mediated responses with enhanced expression of TNF-α, IFN-γ, IL-1β, IL-12, IL-6, IL-10 and IL-17^61^. In this regard, Winnie 10 week colon explants showed prominently significant increase in IL-1β, IL1-α, IL-18, TNF-α, IFN-γ, and MIP1a when compared to C57BL/6 (Fig. 6a–f). This establishes our experimental model 10 week old Winnie, as a clinically relevant model of chronic ulcerative colitis. Importantly our results demonstrated that MCC950 treatment significantly reduced IL-1β, IL-1α, IL17, IL6, IFN-γ, TNF-α and MIP1a in the colitis colon (Fig. 6a–f)). Similarly treatment with MCC950 has been reported to have reduced proinflammatory cytokines and chemokines in other inflammatory diseases such as Influenza A virus infection^33^, in renal inflammation^30^ and a dermal inflammation model^24^. MIP1a (CCL3), is a chemokine that attracts proinflammatory cytokine production and is particularly alleviated in Winnie distal colon. However after oral treatment with MCC950 the MIP1-a levels were significantly lowered. Increased generation of Nitric Oxide leads to excessive production of reactive nitrogen species resulting in infiltration of inflammatory cells and intestinal damage^62^. MCC950 treatment significantly reduced nitrite which is an index for Nitric Oxide production significantly in Winnie proximal and distal colon.

From these results we can conclude that MCC950 inhibition of NLRP3 inflammasome has indirectly suppressed the activation of infiltrating macrophages by inhibiting the release of pro-inflammatory cytokines, chemokine immunomodulators and Nitric Oxide that contributed to the chronic inflammatory process in the Winnie colon. MCC950 is absorbed into the blood stream and is cleared within a window of time. Initially it will be efficacious in the reduction of IL-1β, however proinflammatory cytokines measured in blood plasma taken after 24 hours of treatment did not show any effect on systemic cytokines. This suggest that MCC950 did not have a prolonged adverse systemic effect but its potency on reducing proinflammatory cytokines was significant at the colonic tissue.

We investigated the possibility of MCC950 and glyburide target to be the NEK7-NLRP3 interaction. Shi *et al*. has shown that NEK7 phosphorylation enhances its binding to NLRP3 and promotes inflammasome activation^27^. Our results showed no difference in the level of phosphorylated NEK7 between the untreated and MCC950 or glyburide treated samples. However the IL-1β levels were suppressed by the inhibitors conceding that they successfully blocked the activation of NLRP3 activation. This result suggest that the inhibitory target is not NEK7-NLRP3 but is downstream of this interaction.

The role of NLRP3 inflammasome in colitis is controversial. Genetic ablation of genes of NLRP3 components are predisposed to colitis and colorectal cancer^48^. However, hyper activation of NLRP3 inflammasome leads to colitis^63^. This stresses the need for careful investigation of temporal therapeutic strategies in different disease phases in clinically relevant age appropriate experimental models. The findings from Tate *et al*. on MCC950 treatment for influenza A virus shows that blockade of NLRP3 is detrimental at early stage of disease while protective at late stage of disease^33^. Interestingly our results show that MCC950 is therapeutic in ulcerative colitis at chronic phase of the disease. Further studies are needed to look at emerging colitis to choose the optimal clinical treatment point of MCC950 for ulcerative colitis.

## Conclusion

In conclusion, our results collectively suggest that the small molecule MCC950 paves the way for a novel therapeutic strategy in ulcerative colitis. Our results show, for the first time, the contribution of anti-inflammatory effects resulting exclusively from inhibition of canonical and non-canonical NLRP3 inflammasome activation in colitis. Moreover, the ability of MCC950 to suppress both translational and transcriptional IL-1β and IL-18 of canonical and noncanonical NLRP3 inflammasome in the colon may be promising in inflammatory intestinal diseases other than ulcerative colitis. Nevertheless, the detailed mechanism of the pharmacological target of how MCC950 inhibits the activation of NLRP3 inflammasome needs to be explored in a future study.

## Methods

### Animals

All animal experiments were approved by the Animal Ethics Committee of the University of Tasmania (Ethics approval number: A16166) and conducted in accordance with the Australian Code of Practice for Care and Use of Animals for Scientific Purposes (8th Edition 2013). Mice were housed in a temperature-controlled environment with a 12-hour day/night light cycle. Individual body weights were assessed daily over an initial acclimation period of 7 days. All mice had access to radiation-sterilised rodent feed (Barastoc Rat and Mouse, Ridley AgProducts, Australia) and autoclaved tap water for drinking ad libitum during experiments. All efforts were made to minimize animals’ suffering and to reduce the number of animals used.

### Explant Culture

Mice were euthanized by CO_2_ asphyxiation.The colons were dissected and removed from C57BL/6 and Winnie mice (n = 5, 12 weeks). The colon was opened and the faecal matter removed and cut in half lengthways, sectioned into distal and proximal tissue and weighed. Tissues are washed in PBS (P3813 Sigma) containing 1% penicillin/streptomycin (10000 U/ml) (1% P/S) (Gibco 15140122) three times. Equivalent amount of tissue were placed in a 24 well cell culture plate in growth media RPMI 1640 supplemented with 10% FCS, 1% P/S. The tissues were stimulated with 10 ng/ml Lipopolysaccharide (LPS) from *Escherichia coli* serotype EH100 (ra) TLRgrad for 2 hours. The medium was removed and replaced with serum-free medium (SFM) containing MCC950 (0.001–10 μM) (Adipogen), glyburide (200 μM) (Sigma-Aldrich) and incubated for 24 hours at 37 °C in a moist atmosphere of 5% CO_2_. After which the supernatants were removed and centrifuged at 12,000 g at 4 °C for 15 min and stored at −80 °C for cytokine analysis. Tissue was stored in RIPA buffer with protease inhibitor to be analysed by western blot. Supernatants were assayed for cytokine levels by ELISA kits according to the manufacturer’s instructions IL-1β (DuoSet, R&D Systems) IL10 (BMS614-2 Invitrogen), TNF-α (KMC3011 Invitrogen) and concentrations were normalized to the weight of the explants.

### Isolation of murine macrophages

Mice were euthanized by CO_2_ asphyxiation. Intraperitoneal (IP) macrophages were isolated from the peritoneal cavity of C57BL/6 and Winnie mice (n = 4, 12 weeks) by injection of 10 ml of PBS. After 30 seconds of abdominal massaging, peritoneal lavage was performed. Collected peritoneal lavage was washed twice in PBS and plated in 6 well plates suspended in RPMI-1640 (Gibco 11875093) medium containing 10% FCS (Gibco,10437-028) 1% P/S for two hours. Non adherent cells were removed by washing the plate twice with PBS. The adherent macrophages were analysed in subsequent experiments.

Bone marrow derived macrophages (BMDM) cells were isolated from tibiae and femurs of C57/BL6 mice and Winnie mice (n = 4, 12 weeks) and cultured suspended in RPMI-1640 (Gibco 11875093) medium containing 10%FCS (Gibco, 10437-028) 1% P/S and 10 ng/ml human macrophage colony-stimulating factor (M-CSF) (Miltenyi Biotec). Culture medium was exchanged every 3 days. Under these conditions, an adherent macrophage monolayer was obtained at 7–8 days. Cells were harvested and seeded on 6-well plates. After culturing for 6 hours without M-CSF, the cells were used for the experiments as BMDM.

For the isolation of Mesenteric lymph node (MLN) macrophages the peritoneal cavity of Winnie (n = 4, 12 weeks) was opened and the gut was taken out so that the MLN were visible. The MLN were excised and placed in chilled PBS. To generate a single cell suspension, the MLN were placed on a sterile 70 μm nylon mesh cell strainer (Fisher brand 22-363-548) and was mechanically disrupted into the mesh using the base of a plunger from a 1 cc syringe. Cells were washed in PBS containing 1% FBS. Cell suspension was decanted through a second 70 μm cell strainer to remove any remaining cellular aggregates or tissue debris. Cells were subjected to gentle centrifugation at 500 g for 5 min. Supernatant was decanted and cells re-suspended and cultured in RPMI-1640 medium containing 10% FCS and 1% P/S till an adherent macrophage monolayer was obtained.

Single cell LPMC suspensions were prepared from 12 week old Winnie n = 12 mice. The colons were dissected carefully and washed with ice-cold PBS and cut in to small pieces. Fragments were placed in HBSS containing 5 mM EDTA(sigma) and 1 mM DTT (sigma) 37 °C for 40 mins with gentle shaking to remove the epithelial layer. The colon segments were then digested in PBS containing 0.5 mg/mL collagenase (Sigma), 0.5 mg/mL DNaseI (Roche) and 3 mg/mL Dispase II (Roche) at 37 °C, at slow rotation, for 1.5 hours. Supernatants were collected by filtering through a 70 μm cell strainer. Filtered cells were layered on a 40/80 Percoll gradient and centrifuged at 1000 × g for 20 min. The separated LPMCs were washed twice, and re-suspended and cultured in RPMI-1640 medium containing 10% FCS and 1% P/S.

### Cell viability assay

Cell viability of BMDMs was measured using alamarBlue® reagent (ThermoFisher Scientific). Briefly, 1 × 10^5^ of BMDMs were seeded into each well of a 96-well plate. The following day, the overnight medium was replaced with serum-free media for 12 hours. Cells were then stimulated with different concentrations of MCC950 (0.001, 0.01, 0.1, 1 μM) for 24 h. 10 μl of alamarBlue® reagent was added directly to cells in each well and incubated for 4 h at 37 °C. Then, absorbance at 570 nm was measured. Experiments were repeated three times.

### Inflammasome activation assays

We seeded BMDMs, IPS and MLNs at 5 × 10^5^/ml and LPMCs at 1 × 10^5^/ml in 96-well plates. The following day, the overnight medium was replaced and cells were stimulated with 10 ng/ml LPS from *Escherichia coli* serotype EH100 (ra) TLRgrade (Alexis Biochemicals) for 3 hours. The medium was removed and replaced with SFM containing MCC950 (0.01 μM), glyburide (200 μM) (Sigma-Aldrich), Cells were then stimulated with the following inflammasome activators: 5 mM adenosine 5′-triphosphate disodium salt hydrate (ATP) (sigma) (1 hour), and 10 μM Nigericin (Invivogen) (1 hour). Supernatants were removed and analysed using ELISA kits according to the manufacturer’s instructions IL-1β (DuoSet, R&D Systems), TNF-α (KMC3011 Invitrogen).

### Western blotting

Cell lysates were prepared by direct lysis in 50 μl of 2× Laemmli sample buffer. The protein content of supernatants was concentrated using StrataClean resin (Agilent Technologies) according to the manufacturer’s instructions. The protein samples were resolved on 4–20% mini-protean precast SDS-PAGE gels (Biorad) and transferred onto polyvinylidene difluoride membrane using a wet-transfer system. Membranes were blocked in 5% (wt/vol) dried milk in TBS-T (50 mM Tris/HCL, pH 7.6, 150 mM NaCl and 0.1% (vol/vol) Tween-20) for 1 hour at room temperature. Membranes were incubated with primary antibody diluted in 5% (wt/vol) dried milk in TBS-T and then with the appropriate horseradish peroxidase (HRP)-conjugated secondary antibody diluted in 5% (wt/vol) dried milk in TBS-T for 1 hour. Membranes were developed using SuperSignal West Pico chemiluminescent substrate (Thermo Fisher Scientific). Membranes were stripped using Restore PLUS western blot stripping buffer (Thermo Fisher Scientific) before being re-probed.

Primary antibodies used were ASC antibody (AL177) (1 in 1,000) (Enzo Life Sciences); β-actin; mouse caspase-1 p10 (sc-514) (1:1,000), mouse IL-1β (NB600–633) (1:1,000) and NLRP3 antibody (1:1,000) (NBP2-12446SS). Secondary HRP-conjugated antibodies used were, anti-rabbit IgG and (1:5000) (sc-2030).

### *In vivo* oral administration of MCC950

Seven week-old Winnie mice (homozygous Muc2 mutant; C57BL6/J background) n = 20 of both sexes average weight 18 g were obtained from the University of Tasmania animal breeding facility. Mice were randomly divided into two groups. MCC950 used in *in vivo* experiment was a gift from Avril Robertson.MCC950 treatment group: (n = 10) and control group (n = 10). Treatment group mice were fed 1 g of freshly made chow mash (chow powder blended with water) mixed with 40 mg/kg MCC950 daily. Control mice received the formulation vehicle PBS in 1 g chow mash. The mice were single-caged throughout the experiment to ascertain the defined daily intake of MCC950 from prepared chow mash. Mice were sacrificed on day 21.

### Clinical scoring and histological analysis

Bodyweight, stool consistency and the presence of gross blood in stool and at the anus were observed every day. Stool was collected from individual mice and tested for the presence of blood using Hemoccult II slides (Beckman Coulter Inc., California, USA). The disease activity index (DAI) was calculated by assigning well-established and validated scores^64^. Briefly, the following parameters were used for calculation: a) Stool consistency (0 points = normal, 1points = soft but formed, 2 points = loose stool, 3 points = watery stool) b) blood in stool (0 points = no bleeding, 1 point = Hemoccult+, 2 points = visual blood, 3 points = gross bleeding).

At day 21 following treatment, animals were sacrificed by CO_2_ asphyxiation. The colon from the caecum to the anus was removed. The length of the colon from ileocaecal junction to the rectum was recorded. The colon was subsequently opened along its longitudinal axis and the luminal contents were removed prior to weighing the organ. The colon was bisected longitudinally and one half was prepared using the Swiss roll technique^65^, whereas the remaining colonic tissue was dissected and snap-frozen for molecular analyses. Swiss rolls underwent 24 h fixation in 10% (v/v) neutral-buffered formalin. Swiss rolls were subsequently transferred to 70% ethanol prior to progressive dehydration, clearing and infiltration with HistoPrep paraffin wax (Fisher Scientific, Philadelphia, PA, United States). They were then embedded in wax and 5 μm sections were cut using a rotary microtome. Sections were stained with haematoxylin and eosin Y (H&E; HD Scientific, Sydney, Australia). Slides stained with H&E were evaluated for inflammatory features. Histological inflammation was graded in a blinded fashion by RF and APP based on previously used criteria^66^. Briefly, frequency of lamina propria neutrophils graded 0–2, frequency of crypt abscesses graded 0–2, crypt architectural distortion was graded 0–2, extent of surface damage graded 0–2, goblet cell depletion graded 0–2. The inflammation score for each individual region (distal, middle and proximal colon) was derived from the sum of the score for each of the aforementioned criteria.

### Cytokine measurements

Serum was collected from blood drawn by cardiac puncture at the end of the treatment. Explants from the proximal and distal colons of treatment and control groups (n = 3) were cultured overnight in RPMI 1640. Culture supernatants were measured for Nitrite by the Griess reaction method (Sigma G4410) as an index of Nitric Oxide generation.

Cytokine concentrations in neat culture supernatants and serum were determined using mouse Bio-Plex mouse cytokine 23-plex panel kit (Bio-Rad #M60009RDPD) and analysed using Luminex 200 (Bio-Rad) and Bio-Plex Manager software (Bio-Rad). IL18 was determined by 5 times diluted supernatant measured by a mouse IL18 ELISA kit (7625, R&D Systems). The most significantly altered cytokines are presented as pg per g of tissue.

### RNA extraction and RT-PCR

Colonic tissue was homogenised using rotorstator generator probes (Omni Scientific) and RNA extracted using the RNeasy Mini spin column kit (Qiagen, Melbourne, Australia) according to the manufacturer’s instructions. Integrity and concentration of extracted RNA was assessed using Eppendorf Biophotometer. Complementary DNA (cDNA) was synthesised from RNA samples using the iScript gDNA clear cDNA synthesis kit (Bio-Rad) using reaction conditions suggested by the manufacturer. 100 ng of cDNA from each sample was added to a PCR reaction including TaqMan Fast Master Mix (Applied Biosystems) and a single gene-specific TaqMan probe/primer set. IL-1β, Assay ID: Mm00434228_m1 and IL18 Assay ID: Mm00434226_m1.

Thermal cycling was performed using a StepOnePlus RT-qPCR instrument (Applied Biosystems). Gene expression was quantified using the comparative (ΔΔCT) method where the threshold cycle (CT) for each gene was normalised to reference gene *Gapdh*. Relative gene expression in the MCC950 treated animals was presented as 2^−ΔCT^.

### NEK7 phosphorylation state analysis by Phos-tag SDS-PAGE

We seeded J774A.1 at 1 × 10^5^/ml in 6-well plates. The following day, the overnight medium was replaced and cells were stimulated with 100 ng/ml LPS for 3 hours. The medium was removed and replaced with SFM containing MCC950 (1 μM) and glyburide (200 μM) for 30 min or no treatment with SFM. Cells were then stimulated with 5 mM ATP for 30 min. Supernatants were removed and analysed using ELISA kits according to the manufacturer’s instructions IL-1β (DuoSet, R&D Systems). Cell lysates from three wells were pooled in 200 ul of RIPA buffer (R0278 Sigma) with phosphate inhibitor (Roche). Contaminants were removed in protein samples by TCA precipitation. Samples mixed in 2× Laemmli sample buffer and run in Phos-tag SDS-PAGE (Wako Chemicals) for the separation of phosphorylated proteins according to their degree of phosphorylation. Membranes were developed using SuperSignal West Pico chemiluminescent substrate (Thermo Fisher Scientific). Membranes were stripped using Restore PLUS western blot stripping buffer (Thermo Fisher Scientific) before being re-probed. Primary antibodies used were NEK7 antibody (Ab109433) (1 in 1,000) (Abcam). Secondary HRP-conjugated antibodies used were, anti-rabbit IgG and (1:5000) (sc-2030).

### Statistical analysis

Data are presented as average values ± SEM from multiple individual experiments each carried out in triplicate measurements in a representative experiment. Change in body weight percentage over time was compared using repeated-measures analysis of variance (ANOVA). The statistical significance of the normalised mRNA expression was tested by one sample t-test. Differences in histological scores between anatomical regions were tested post-ANOVA using Tukey’s multiple pairwise comparisons test. Statistical analyses were done using a nonparametric unpaired two-tailed *t*-test, for two groups study. The data were evaluated with one-way analysis of variance (ANOVA) for 3 groups study and confirmed using Tukey’s test for multiple comparisons using Prism software (GraphPad). Data were considered significant when *P* ≤ 0.05 (*), *P* ≤ 0.01 (**), *P* ≤ 0.001 (***) or *P* ≤ 0.0001 (****).

### Data availability

The datasets supporting the conclusions of this study are included within this published article and its supplementary file.

## Electronic supplementary material

Supplementary Information
