# Supplementary material for: MCC950, a specific small molecule inhibitor of NLRP3 inflammasome attenuates colonic inflammation in spontaneous colitis mice
Source: Sci Rep. 2018 Jun 5;8:8618. doi: 10.1038/s41598-018-26775-w (PMC5988655; doi:10.1038/s41598-018-26775-w)

Supplementary data to:

**MCC950, a specific small molecule inhibitor of NLRP3 inflammasome attenuates colonic inflammation in spontaneous colitis mice**

Agampodi Promoda Perera^1^, Ruchira Fernando^2^, Tanvi Shinde^1^, Rohit Gundamaraju^1^, Benjamin Southam^1^, Sukhwinder Singh Sohal^1^, Avril A.B Robertson^3^, Kate Schroder^3^,

Dale Kunde^1^, Rajaraman Eri^1^

^1^School of Health Sciences, University of Tasmania, Launceston, TAS, Australia,  ^2^Department of Pathology, Launceston General Hospital, Launceston, TAS Australia, ^3^Institute for Molecular Bioscience, University of Queensland, St Lucia, QLD, Australia

**SUPPLEMENTARY FIGURE S1. AlamarBlue® Cell viability assay results.** Data represents the mean viability ± SEM from duplicate determinations and representative of three independent experiments.

**SUPPLEMENTARY FIGURE S2. Uncropped Western blot images (a-e)**, this figure represents full-length Western blot images of original Figure 1 (e).

a.


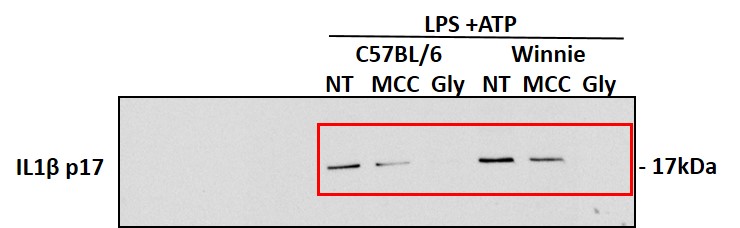


b.


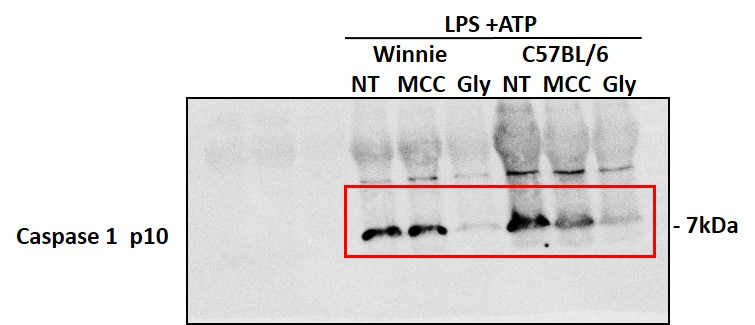


c.


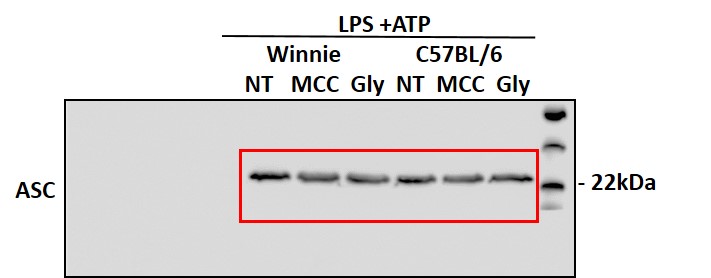


d.


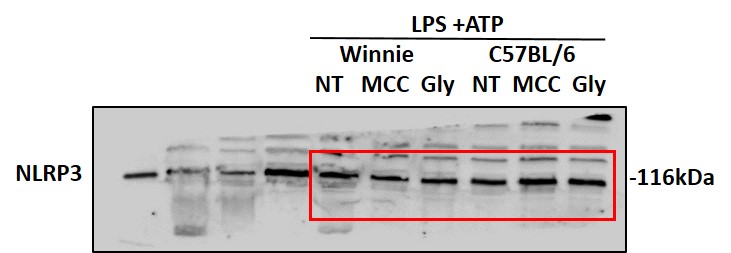


e


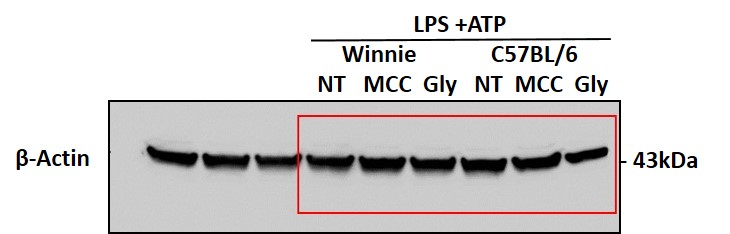


**SUPPLEMENTARY FIGURE S3. Uncropped Western blot** images (a-e). This figure represents full-length Western blot images of original Figure 2 (d)

a


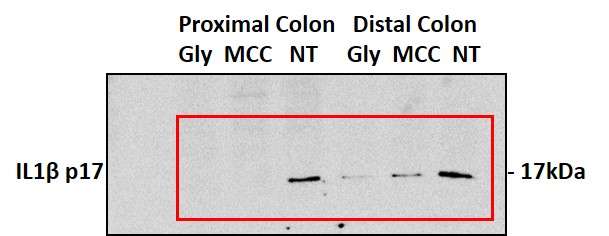


b


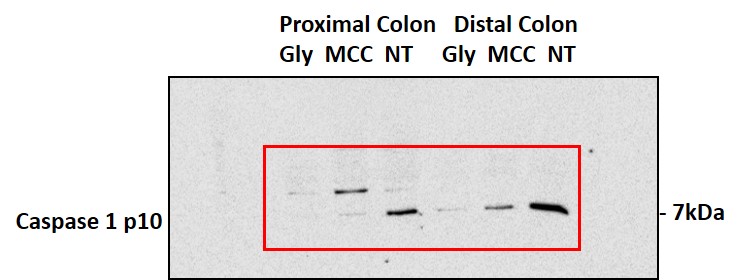


c


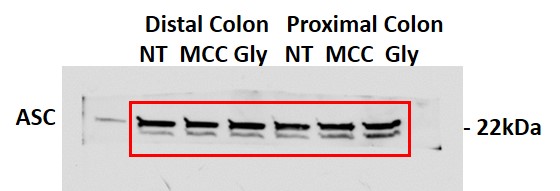


d


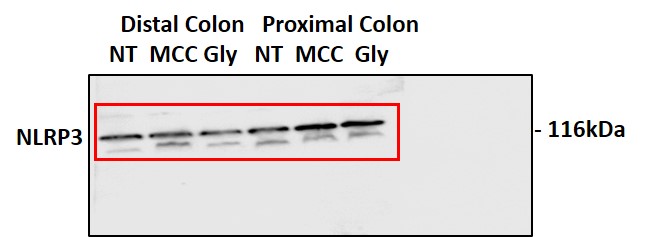


e


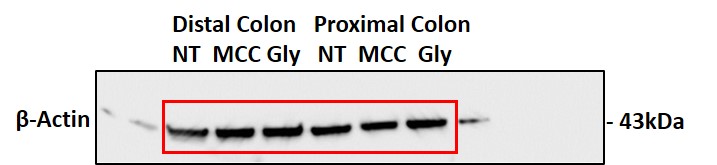


**SUPPLEMENTARY FIGURE S4. Uncropped Western blot images (a-c).** This figure represents full-length Western blot images of original Figure 7 (a).

a


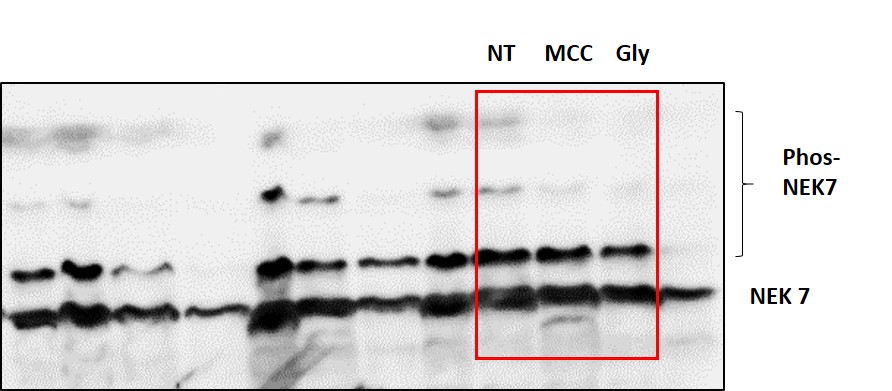


b


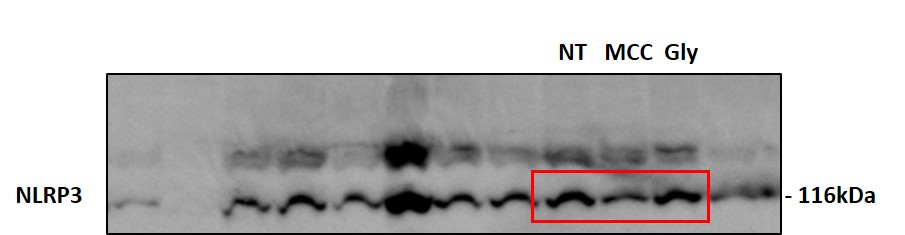


c


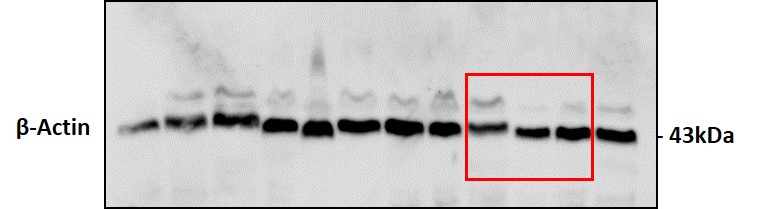

Supplement: Supplementary file 1 — Supplementary Information [file 41598_2018_26775_MOESM1_ESM.doc]
